# A soft computing scheme incorporating ANN and MOV energy in fault detection, classification and distance estimation of EHV transmission line with FSC

**DOI:** 10.1186/s40064-016-3533-2

**Published:** 2016-10-21

**Authors:** Piyush Khadke, Nita Patne, Arvind Singh, Gulab Shinde

**Affiliations:** 1 Electrical Engineering Department, Visvesvaraya National Institute of Technology, Shankar Nagar, Nagpur, 440010 Maharashtra India; 2 Power Grid Corporation of India Ltd., 1200/765/400/220 kV Wardha Substation of Power Grid Corporation of India Ltd., Deoli, Wardha, 442101 Maharashtra India

**Keywords:** Artificial neural network, Transmission lines, Power system faults, Fault location

## Abstract

In this article, a novel and accurate scheme for fault detection, classification and fault distance estimation for a fixed series compensated transmission line is proposed. The proposed scheme is based on artificial neural network (ANN) and metal oxide varistor (MOV) energy, employing Levenberg–Marquardt training algorithm. The novelty of this scheme is the use of MOV energy signals of fixed series capacitors (FSC) as input to train the ANN. Such approach has never been used in any earlier fault analysis algorithms in the last few decades. Proposed scheme uses only single end measurement energy signals of MOV in all the 3 phases over one cycle duration from the occurrence of a fault. Thereafter, these MOV energy signals are fed as input to ANN for fault distance estimation. Feasibility and reliability of the proposed scheme have been evaluated for all ten types of fault in test power system model at different fault inception angles over numerous fault locations. Real transmission system parameters of 3-phase 400 kV Wardha–Aurangabad transmission line (400 km) with 40 % FSC at Power Grid Wardha Substation, India is considered for this research. Extensive simulation experiments show that the proposed scheme provides quite accurate results which demonstrate complete protection scheme with high accuracy, simplicity and robustness.

## Background

Modern power systems with ever increasing power demands require power with satisfactory standards of quality and economy with feasible cost cutting measures (Hor et al. 2010; Nurdin et al. 2012). In developing countries, the optimized use of transmission system investments is very important. Practical application of FSC has been found it as the suitable choice not only to achieve the desired power increment, but also to stabilize the two interconnected strong networks by reducing the connecting line impedance of the given corridor’s transmission capacity (Oliveira 2008). In addition to this, FSC is the simplest and cost-effective solution of increasing the power transfer capability of a power system that has long (250 km or more) transmission lines (Oliveira 2008).

Estimation of the impedance to the fault point is influenced by the series compensation (Vyas et al. 2014a, b). Series compensation affects the fault location estimation in an unpredictable manner. Most of the reactance-based topologies suffer from mal-operation under reach due to DC offset current. Also, these schemes require measurement from both ends if the transmission line is having two end sources. In addition, the problem gets worse if the overvoltage protection of a series capacitor starts to operate due to high fault currents introducing nonlinearities in the measurement (Hosny and Safiuddin 2009). All of these nonlinear behavior due to series capacitor and its protection contributes to the distortion in phase voltage and line current waveforms. So, false tripping and mal-operation of circuit breakers is dominant due to series compensation. Hence conventional impedance based protection systems are most likely to malfunction (Hosny and Safiuddin 2009).

Fault classification and fault location estimation increases system stability, reliability and availability. Accurate location of faults on overhead transmission lines for inspection maintenance purposes is of vital importance for expediting service restoration to reduce service outage time and operating costs. Accurate fault analysis with location estimation will surely improve overall transient stability and reduce the switching overvoltage in the power system. Therefore, the fault type classification and fault distance estimation have become very important aspects of protection of series compensated transmission line.

MOV energy signals are never implemented before in any of the earlier conventional fault classification and location estimation algorithms. In order to find some new solution, authors have considered a new measurement scheme on FSC side rather than conventional line side voltage and current measurement.

A bibliographical survey of relevant background, effect of series compensation on transmission line protection and protection efforts for series compensated line is presented in Vyas et al. (2014a). Various fault-location algorithms for series-compensated lines have been developed so far. They apply one-end (Hosny and Safiuddin 2009; Ray 2014; Ray et al. 2013; Parikh et al. 2008; Abdelaziz et al. 2005; Vyas et al. 2014b; Moravej et al. 2012) and two-end measurements (Izykowski et al. 2011; Rubeena et al. 2014; Ahsaee and Sadeh 2011; Kang et al. 2015; Eldin 2010; Hussain and Osman 2014; Al-Dabbagh and Kapuduwage 2005; Yusuff et al. 2011; Ma et al. 2015; Abdelaziz et al. 2013) for two-terminal lines. In general, the impedance-based approach is the mostly applied. Multilayer perceptron neural networks (MLPNN) based scheme with two neural networks to address fault classification and location is proposed in Hosny and Safiuddin (2009). Fault location by extreme learning machine with genetic algorithm based feature selection method is depicted in Ray (2014). Wavelet transform (WT) and wavelet packet transform (WPT) combining ANN method for fault distance estimation has been considered in Ray et al. (2013) while a combined wavelet-support vector machine (SVM) technique for fault zone identification in series compensated transmission line has been investigated in Parikh et al. (2008), Yusuff et al. (2011). Two approaches based on travelling waves and two level ANN for fault type classification and faulted phase selection of series compensated transmission lines have been proposed in Abdelaziz et al. (2005). A new approach based on hyperbolic S-transform for extracting useful features from the input signals and support vector regression for fault location is presented in Moravej et al. (2012). A more general case of unsynchronized measurements utilizing two subroutines for locating a fault has been presented in Izykowski et al. (2011). Direct Prony analysis and four-cycle discrete Fourier transform algorithm based new offline technique is proposed for accurate estimation of fault current and voltage phasors of the series compensated transmission system in Rubeena et al. (2014). In Eldin (2010), a new system with wavelet MRA coefficients for fault detection and classification and adaptive neuro-fuzzy inference system (ANFIS) to obtain accurate fault location has been studied. A generalized fault loop method using pre-fault PMU measurements is considered in algorithm (Al-Mohammed and Abido 2014). A fault direction estimation technique for a series compensated line using phase change in positive-sequence current and magnitude change in the positive-sequence voltage at fault is provided in Jena and Pradhan (2010). Detailed analysis of a new method for fault event detection and optimum relay coordination in wind farm using genetic algorithm is given in Perven et al. (2015). A new concept of integrated impedance based intelligent relaying for transmission line with TCSC is introduced in Jena and Samantaray (2015). The technique in Moravej et al. (2011) presents a new combined S-Transform and Logistic Model Tree techniques for fault classification and fault section identification in transmission system with TCSC. Both schemes in Jena and Samantaray (2015), Moravej et al. (2011) lack to give fault distance estimation. In Ray (2014), Ray et al. (2013), post fault one cycle voltage and current signals have been taken while in Parikh et al. (2008), Ma et al. (2015) post fault one cycle current signals have been considered. Post fault half cycle window signals are used in Vyas et al. (2014a, b), Yusuff et al. (2011).

ANN is powerful in pattern recognition, classification and generalization tool (Vyas et al. 2014a, b). Off-line data training is very useful feature of ANN. Immunity to noise, robustness and tolerance to fault are a few of the advantages of ANN over other pattern recognition tools. Nonlinearity and variations in system parameters will not seriously affect an ANN-based relay decision. So, various ANN-based algorithms have been investigated and implemented in power systems in recent years Jain et al. (2009).

**Fig. 1:**
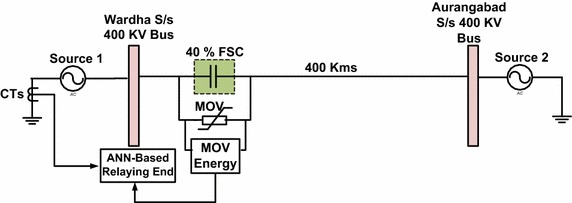
Schematic of series compensated transmission line with FSC

In this research work, an equivalent model of 400 kV Wardha–Aurangabad line (400 km) which is under construction with end buses and 40 % fixed series capacitors at Wardha Substation end, i.e. relaying end with protection scheme has been developed based on the real time parameters of the actual installed system. This FSC is being installed at 1200/765/400/220 kV Wardha Substation, Power Grid, Western Region (WR)—I, India. Figure 1 shows the considered equivalent schematic of series compensated transmission line with two 400 kV end buses as described above.

This paper presents the application of ANN for fault distance location in a double end fed single circuit transmission line. All the ten types of internal shunt faults using only one terminal data, i.e. mainly MOV energy signals from relaying end have been considered. These MOV energy signals are taken over only one cycle window from the inception of fault which makes this scheme rapid. The effects of varying fault type, fault location and fault inception angle have been studied.

At present, quadrilateral characteristic distance relays are used for distance protection of 400 kV fixed series compensated transmission line and is reported to perform less accurate in estimating fault location distance. Line side voltage and current measurement get affected abruptly in series compensated line, whereas, MOV energy signals are not affected as compared to impedance measurement signals used in other distance protection schemes due to fixed series compensation.

The performance of the proposed scheme has been investigated by a number of offline tests for internal faults. Percent absolute error criterion is chosen to evaluate the proposed method performance. The simulation results show that all the ten types of internal faults can be correctly classified and located after one cycle from the inception of fault. In addition, the proposed scheme does not require a communication link to recover the remote end data. Such comprehensive work has not been reported earlier for fault classification and fault location estimation of fixed series compensated line using MOV energy signals of fixed series capacitors (FSC) as input to train the ANN with soft computing paradigm.

This paper is structured into 7 main sections; the first section is an introduction. In the “Working philosophy of FSC” section we discuss the basic protection scheme of FSC and in “System configuration and modeling” section system configuration and modeling in Matlab/Simulink with above mentioned real time system parameters is explained. Section “Implementation of ANN” focuses on ANN and learning rule implemented in this novel scheme while “Proposed algorithm” section introduces the detailed explanation of the proposed scheme of fault classification and fault location estimation with complete algorithm. Also, the details of training the ANN with MOV energy signals at various fault conditions to get the minimum error is explained in “Proposed algorithm” section. Section “Performance evaluation and results” presents the performance evaluation and simulation results of the proposed scheme in terms of percent absolute error. Finally, “Conclusions” section clarifies the extracted conclusions with this new superior proposed protection scheme of series compensated transmission line.

## Working philosophy of FSC

Fixed series compensation has been utilized for many years with excellent results in AC power transmission. As show in Eqs. (1) and (2), series compensated transmission system will increase the active power transmission capability over the given corridor without affecting angular or voltage stability (Nurdin et al. 2012).

1 $${\text{Power}}\;{\text{transmitted}}\;{\text{without}}\;{\text{FSC}} = Vs \times Vr \times sin\delta /X_{l}$$

2 $${\text{Power}}\;{\text{transmitted}}\;{\text{with}}\;{\text{FSC}} = Vs \times Vr \times sin\delta /\left( {X_{l} - Xc} \right)$$

3 $$V = f\left( {P,Q} \right)$$

4 $$Qc = 3X_{c} I^{2}$$

where, *δ* = Angular difference between voltages, *Vs* = Sending end voltage, *Vr* = Receiving end voltage, *X*_*l*_ = Line reactance, *Xc* = Series capacitor reactance, *P*, *Q* = Received active and reactive powers respectively, *Q*_*c*_ = Reactive power of series capacitor

**Fig. 2:**
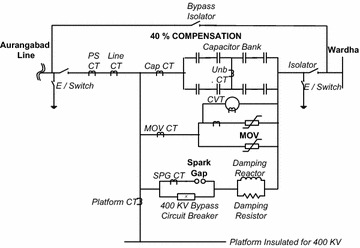
Single-line scheme of actual installation of 400 kV FSC

In Fig. 2, single-line scheme of actual installation of 400 kV FSC is shown. The series capacitor banks in FSC are protected with MOV and forced triggered spark gap protection (IEEE Standards 2013; Haddad et al. 2001; Ahmed et al. 2012). In case of a fault in the transmission line, high short circuit current will flow also through the capacitor bank which will increase the voltage across the capacitors. It is not economically feasible to design the capacitors to withstand very high voltages. Therefore the MOV having Zinc Oxide (ZnO) discs are connected in parallel with the capacitor bank having highly non linear Voltage vs. Current characteristics. As long as the voltage across the capacitor is below the protective level, the MOV presents a very high resistance. Having the voltage across the capacitor terminals greater than the protective level, the MOV resistance becomes very low, and in turn, diverting a significant portion of the fault current away from the capacitor.

In case of external fault (fault outside the line section, where the series capacitor is located), the MOV is so designed that it will limit the voltage to the protective voltage level, keeping capacitor banks in circuit until the line circuit breakers in the external line will clear the fault.

In case of internal fault (fault inside the same line section, where the series capacitor bank is located), the forced triggered spark gap will bypass the MOV and the capacitor bank only when the fault current crosses the maximum current rating of the capacitor units (i.e. 3000 A in this case) and MOV dissipation energy level crosses its threshold limit (i.e. 15 MJ) thus protecting them. At the same time, the closing command is given to the bypass circuit breaker (BPCB) in order to protect and extinguish the spark gap.

The damping circuit helps to limit and dampen the discharge current of the capacitor bank in case the spark gap operates or the BPCB is closed. Current transformers (CTs) are provided at different locations to measure the current for different protection systems. The two Isolators are used to connect the platform and platform equipments to the line or to isolate the platform and platform equipments from the line. The earth switches are used for earthing the platform and platform equipments, as soon as the platform is completely isolated from the line.

## System configuration and modeling

Feasibility and reliability of the proposed scheme are investigated using fault data set of 400 kV power system simulated in Matlab/Simulink software. Actual 400 kV series compensated Wardha–Aurangabad transmission line (400 km) is considered for power system modeling. A two bus three phase 400 kV, 50 Hz transmission system as shown in Fig. 2, has been simulated for the study of fault classification and location problem. The power system comprises two sources, 400 km transmission line and FSC with its associated protecting components. The transmission line is simulated using a distributed parameter line model. A discrete sampling frequency of 2 kHz is used in simulation. The FSC installation is located at the Wardha substation end of the transmission line. 40 % series compensation is achieved with this FSC. The transmission line parameters and other real time data at bus used for simulation are given in the “Appendix” section. Table 1 gives ratings of various components of FSC installed at Wardha Substation.

**Table 1:** Ratings of various components of FSC

Sl. no	Description	Rating
1	Fixed compensation capacitor bank	43.04 Ω, 73.96 μF, 3000 A, 387.36 MVAR/phase
2	MOV for capacitor bank	MCOV = 130 kV rms, *E* = 15 MJ/phase
3	Spark gap	FOV = 400 kV_p_
4	Damping resistor	10 Ω
5	Damping reactor	700 μH, 3000 A
6	By-pass circuit breaker	400 kV, 3 phase, SF6, 3150 A

*MCOV* maximum continuous operating voltage, *FOV* flash over voltage

Total line reactance is *X*_*l*_ = 107.6 Ω. The capacitance of FSC, *C* = 73.96 μF and capacitive reactance of FSC is *Xc* = 43.04 Ω which is 40 % of Wardha–Aurangabad total line reactance. The capacitor bank is designed for maximum current rating of 3000 A. The MCOV of MOV is kept 130 kV in this case. Since the MOV is a non-linear resistive element and it has an energy dissipation limit, it is protected against excessive heat by an overload protection. The overload protection calculates the energy absorbed by the MOV and triggers a parallel air gap if the energy exceeds a threshold value i.e. 15 MJ used in this study.

The MOV energy in the model is calculated in Eq. (5) over one cycle after inception of fault as,

5 $$MOV\,Energy = \smallint \left( {Vmov} \times {Imov} \right)dt$$

where, *Vmov* = Voltage across MOV, *Imov* = By-pass current through MOV.

In practice, these measurements are done with 0.2 accuracy class current and capacitive voltage transformers (CVT) which gives highly accurate measurements and saturation effects of measuring current and voltage transformers can be avoided.

## Implementation of ANN

ANN is a family of models motivated by biological neural networks which can be used to approximate the functions that can depend on a large number of inputs which are generally unknown and random. ANN is an efficient random function approximation tool (Vyas et al. 2014a, b). ANN can be powerfully used in online learning and large data set base applications. After appropriate mapping of inputs and outputs in ANN, the connections will enclose the non-linearity of the desired mapping.

**Fig. 3:**
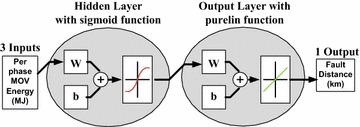
Generalized structure of ANN

The ANN structural design is set by experimenting different network cases and configurations with the number of inputs and the number of hidden layer neurons. A two-layer feed-forward network with sigmoid hidden neurons and linear output neurons is used here in order to solve multi-dimensional mapping problems. Figure 3 gives the general structure of ANN to be used. The input parameter for the ANN is per phase MOV energy, calculated over one cycle post fault. The output parameter of ANN is the fault distance from relaying point. For each type of faults, the number of hidden neurons in the hidden layer is selected based on the performance of the ANN for each case. For all the single line to ground (L–G) faults, the best performance is found with 3 hidden neurons in the hidden layer. For all the double line to ground (L–L–G) faults, 4 hidden neurons provided the best results, for all line to line (L–L) faults, 5 hidden neurons in hidden layer gave best results. Similarly, in case of three phase faults, 7 hidden neurons are found to give best performance. The selection of the number of hidden neurons w.r.t best performance is arrived after testing for numerous cases with increasing number of hidden neurons in hidden layer up to 50. The presented ANN has two layer feed forward network, one hidden layer with sigmoid transfer function and one output layer with linear transfer function purelin as depicted in Fig. 3.

Advantages of ANN over other classifiers are (1) it can be easily implemented in parallel architectures which reduces the processing time compared to other kind of algorithms, attaining comparable results, (2) it is able to obtain non-linear and complex relationships, (3) response is better and (4) it can handle large amount of data sets resulting in easy implementation in a digital relaying system.

It has been proven that approximation of any nonlinear function to arbitrary accuracy can be achieved by backpropagation learning with sufficient hidden layers. This makes backpropagation learning neural network a good investment for system modeling and signal prediction. The back-propagation learning rule is therefore used in perhaps 80–90 % of practical applications. The most suitable training method for the algorithm of this research work is carried on with the Levenberg–Marquardt optimization technique. It is fast and has stable convergence. It has become a standard method for non-linear least-squared problems and is widely adopted in various applications and streams for tackling with real time data-fitting problems.

## Proposed algorithm

Series compensation affects the impedance estimation due to the fault and causes distortion of phase voltage and line current waveforms resulting in the fault location estimation in an unpredictable manner. Whereas, MOV energy signals are not affected as compared to impedance measurement signals used in protection schemes due to fixed series compensation. MOV energy signals are never implemented before in any of the earlier conventional fault classification and location estimation algorithms which make this online scheme distinctive. The use of online MOV energy signals with relaying end neutral current makes this scheme very easy and unique.

**Fig. 4:**
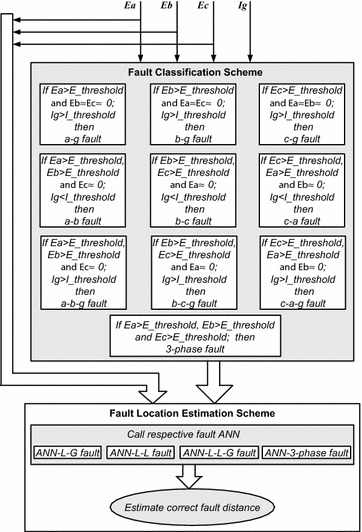
Proposed algorithm

The proposed algorithm consists of two stages, namely fault detection with classification and accurate fault location estimation. Figure 4 shows the main structure of the proposed algorithm.

### Fault detection and classification

Whenever there is an internal fault on the specified line, only respective phase MOV energy increases above threshold. This principle is used for the detection and classification of fault here and is illustrated in Result section. Provision of MOV energy measurement at FSC end is made. The MOV energy per phase is continuously monitored per cycle by the relay and as soon as the energy crosses the specified threshold value, the fault is detected and maximum value of MOV energy over one cycle from that instant is measured. *Ea*, *Eb*, *Ec* and *Ig* are the MOV energy signals per phase and neutral current at relaying end respectively. These signals are assumed to be passed through antialiasing filter. To classify the type of the fault, the magnitudes of these input signals are continuously compared with the predefined respective threshold values (which are given in Table 3) as follows:

1. If the magnitudes of MOV energy in only one phase and *Ig* are greater than the threshold, then the fault is classified as single phase to ground (L–G) fault.
2. If the magnitudes of MOV energy in any two phases and *Ig* are greater than the threshold while the magnitude of MOV energy in third phase is negligible, then the fault is classified as a double phase to ground (L–L–G) fault.
3. If the magnitudes of MOV energy in any two phases are greater than the threshold while the magnitude of MOV energy in third phase is negligible and *Ig* is less than the threshold, then the fault is classified as phase to phase (L–L) fault.
4. If the magnitudes of MOV energy in all the three phases are greater than the threshold simultaneously, then the fault is classified as 3-phase fault.

### Fault location estimation

In this stage, the output of fault classification stage and MOV energy signals are used as input to the respective fault ANN to estimate accurate fault distance from relaying end. Four efficiently trained ANN networks for L–G, L–L, L–L–G and 3-Phase faults respectively are considered. Respective ANN is then activated according to the type of fault which then detects accurate fault location as the output of the proposed scheme as shown in algorithm Fig. 4. If there is an external fault, i.e. fault in another line behind the FSC then the main relay will recognize it as a reverse fault (as followed in industry at present) and the main relay will not let the proposed relay operate for such external fault proper logical coordination assigned. Further the fault point outside the line, it will have less impact on MOV energy and following this convention, the change in the MOV energy will not cross the set threshold value of the proposed algorithm and this relay will not operate for any external fault.

To design the best ANN, it is crucial to train it efficiently and correctly. The training sets are carefully chosen so that all fault conditions, i.e. different FIA and fault locations, are considered. The performance of this soft computation ANN scheme is then tested using random fault conditions in the training set. The approach adopted here is based on the Levenberg–Marquardt (Trainlm) optimization technique. To find the optimum values of the ANN parameters, input signals of *Ea*, *Eb* and *Ec* corresponding to a particular fault condition are fed to the ANN and its output is compared with the desired output corresponding to that fault condition. The ANN is re-trained after each set of new fault conditions.

The expert system database is obtained by extensively simulating the system under normal and fault conditions of a transmission line during the investigation. The inputs are combined and also linked with the output, based on the expert system database to find the accurate ANN output. Accuracy of ANN outputs was also tested with various numbers of hidden layer neurons (up to 50 neurons). Best accuracy is found with 3 neurons in hidden layer for L–G fault, 4 neurons in hidden layer for L–L–G fault, 5 neurons in hidden layer for L–L fault and 7 neurons in hidden layer for 3-phase fault. The various extensive fault conditions considered to train the ANN are explained in Performance evaluation and result section.

## Performance evaluation and results

In order to test the validity of the proposed scheme, the model with the above mentioned system parameters is designed using Matlab/Simulink. Different fault types, such as single line to ground, double line to ground, double line and three-phase faults at different locations in front of the series compensator and different inception angles with realistic fault resistance are extensively investigated. To validate the robustness of the computational intelligent technique in the proposed algorithm, it is tested on a variety of fault cases as given in Table 2 and outputs are checked at random fault conditions for more than 9300 fault cases.

**Table 2:** FSC data generation for fault cases

Sl. no	Variation	Range	Cases
1	Type of faults	a–g, b–g, c–g, a–b–g, b–c–g, a–c–g, a–b, b–c, a–c, a–b–c	10
2	MOV energy	All 3 phases	3
3	Fault locations	10–390 km (in step of 10 km)	39
4	Fault inception angle	0°, 45°, 90°, 135°, 180°, 225°, 270° and 315°	8
	Total cases = 10 × 3 × 39 × 8 = 9360		

In most of the earlier algorithms, only few inception angles are considered. In order to get a more comprehensive vision on the effect of FIA on the proposed scheme, different inception angles over complete cycle are considered as listed in Table 2. For a particular distance, all the MOV energies simulated at above mentioned inception angles are averaged for simplicity. All such MOV energy values at various fault locations are given as input to train respective ANN network accurately. The threshold values used for the proposed scheme are calculated by simulating with severe fault conditions at the other end of relaying bus i.e. very near to 400 kV Aurangabad substation bus. These threshold values found by precise simulations are depicted in Table 3.

**Table 3:** Threshold values

Sl. no	Description	Threshold values
1	MOV energy for L–G fault	0.3 MJ
2	MOV energy for L–L fault	4.2 MJ
3	MOV energy for L–L–G fault	3.5 MJ
4	MOV energy for 3-phase fault	5 MJ
5	*Ig* for L–G fault	2000 A
6	*Ig* for L–L fault	100 A
7	*Ig* for L–L–G fault	1400 A

These threshold values are flexible and can be varied according to various system parameters and requirements of the particular transmission line. Even if there is involvement of ground due to another reason for the short time, threshold MOV energy value will not be crossed and proposed scheme will not treat it as a fault and the same has been checked.

An absolute error criterion is used in assessing the quality of the fault location schemes and the same is adopted in most of the referred work in the literature. So, the performance criterion for evaluating the fault location scheme was selected to be the percentage absolute error. The percentage absolute error is defined as Eq. (6),

6 $$\frac{{\left| {Actual\;fault\;distance - ANN\;fault\;distance} \right|}}{{\left( {Total\;line\;length} \right)}} \times 100$$

Tables 4, 5, 6 and 7 show the ANN estimated fault locations for L–G (a–g), L–L (a–b), L–L–G (a–b–g) and 3-Phase (a–b–c) faults respectively. In Tables 4, 5, 6 and 7, the column with ‘FIA’ heading gives the inception angle at which the fault has been created. The column with ‘Actual Fault Location’ heading gives the fault location where the respective fault has been created on the transmission line in simulations. The column with ‘ANN Fault Location’ heading gives the estimated fault location which is the final output of trained ANN. As we have considered an absolute value for % error calculation in the fault distance estimation, all the values in ‘Abs. error’ column in Tables 4, 5, 6 and 7 are positive (+ sign). The fault distance was calculated from trained ANN which was trained for all types of faults with input of MOV energy. After fault detection and classification, respective fault type ANN was called in the algorithm which estimated the fault location. ANN for L–G fault was trained by one phase MOV energy inputs, ANN for L–L–G and L–L faults were trained with two fault phase MOV energy inputs and ANN for 3-phase fault was trained with three phase’s MOV energy input signals.

**Table 4:** Test results of ANN-based fault locator for a–g (L–G) fault

MOV energy a-ph (MJ)	FIA (°)	Actual fault location (km)	ANN fault location (km)	Abs. error (%)
13.7300	90	20	20.519	0.129
13.4156	45	30	26.235	0.941
13.3172	225	50	46.961	0.759
13.2108	90	100	99.475	0.131
12.7592	0	130	131.865	0.466
10.2576	135	150	150.220	0.055
8.4633	315	170	169.362	0.159
6.4414	135	200	198.982	0.254
4.4918	135	240	238.469	0.383
2.9581	225	280	280.917	0.229
2.1387	45	310	310.097	0.024
1.6873	225	330	328.969	0.259
1.0738	45	360	358.835	0.291
0.7078	315	380	379.548	0.113

**Table 5:** Test results of ANN-based fault locator for a–b (L–L) fault

MOV energy a-ph (MJ)	MOV energy b-ph (MJ)	FIA (°)	Actual fault location (km)	ANN fault location (km)	Abs. error (%)
14.2421	14.0606	45	20	19.110	0.223
13.7765	13.9279	315	30	30.743	0.186
13.5285	13.4685	45	50	47.259	0.685
13.3872	13.3830	0	80	78.854	0.286
13.3412	13.3794	180	100	95.783	1.054
13.2796	13.3006	45	120	118.079	0.480
13.2383	13.1856	0	200	200.987	0.246
13.2290	13.1802	315	210	211.025	0.256
13.1400	13.1790	180	240	244.489	1.122
10.2597	11.2266	0	280	276.632	0.842
8.2746	9.2286	135	310	316.215	1.554
6.8715	7.4042	270	330	332.083	0.521
5.9364	6.4356	90	360	356.125	0.969
5.7576	6.5339	0	380	376.758	0.810

**Table 6:** Test results of ANN-based fault locator for a–b–g (L–L–G) fault

MOV energy a-ph (MJ)	MOV energy b-ph (MJ)	FIA (°)	Actual fault location (km)	ANN fault location (km)	Abs. error (%)
14.3241	14.4362	45	20	23.041	0.760
14.0472	13.7246	315	30	29.135	0.216
13.4605	13.5245	225	50	52.025	0.506
13.3956	13.3809	315	90	87.154	0.711
13.3502	13.3528	45	100	99.118	0.220
13.3489	13.3012	135	120	122.265	0.566
13.2493	13.2646	90	150	149.099	0.225
13.2002	13.2403	90	170	168.850	0.287
13.1720	13.2105	135	200	194.404	1.399
13.1466	13.1619	180	240	242.428	0.606
10.4166	11.4689	45	280	273.366	1.658
8.7512	9.8457	225	310	305.064	1.234
7.4371	8.3886	135	330	330.146	0.036
6.4755	7.6890	0	360	353.889	1.527
5.1643	6.2444	0	380	377.318	0.670

**Table 7:** Test results of ANN-based fault locator for a–b–c (3-phase) fault

MOV energy a-ph (MJ)	MOV energy b-ph (MJ)	MOV energy c-ph (MJ)	FIA (°)	Actual fault location (km)	ANN fault location (km)	Abs. error (%)
14.6019	14.9586	14.0781	45	20	20.767	0.192
13.7642	13.7174	14.0401	90	40	37.319	0.670
13.6367	13.5339	13.8629	270	50	49.129	0.217
13.4283	13.4839	13.3938	225	70	72.997	0.749
13.4112	13.4025	13.3124	90	100	98.142	0.464
13.3317	13.3024	13.2847	0	130	125.238	1.190
13.2924	13.2707	13.2742	270	150	143.380	1.654
13.2509	13.2450	13.2677	180	170	174.765	1.191
13.1822	13.2299	13.2264	225	210	214.327	1.082
10.3153	9.6280	12.5491	315	300	307.514	1.878
7.8287	7.2297	9.8042	135	350	358.165	2.041
7.6658	9.2136	6.2101	45	370	373.314	0.828
7.2897	8.7731	5.8070	45	380	381.924	0.481

MOV energy measured at various locations at different inception angles was given as input to the trained ANN and output was given as soft computation estimated fault location by ANN. Outputs at randomly selected fault inception angles were checked and presented so as to get further clarified vision and more accuracy. The faults were created at 8th cycle. A realistic fault resistance of 0.1 Ω was utilized considering the high severity of the faults and previously recorded real fault resistance values at the Wardha Substation.

Since the proposed scheme employed in this analysis is based on a distributed line model, exact transmission line parameters and results are accurate, this scheme can be implemented in a real fixed series compensated line.

**Fig. 5:**
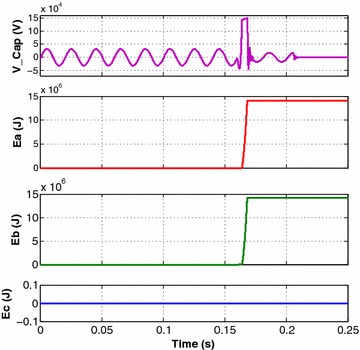
a-Phase capacitor voltage, per phase MOV Energy in for an internal a–b fault

Figure 5 shows simulation results of phase-a capacitor voltage with MOV Energies in a, b and c phases, respectively, for an internal a–b (L–L) fault on 400 kV Wardha–Aurangabad line at 25 km from Wardha substation relaying end. It was seen in Fig. 5, as soon as the internal faults occur, MOV started conducting thus bypassing and protecting the series capacitor banks. For a–b phase to phase fault in Fig. 5, it was seen that the magnitudes of MOV energy in only phase-a and phase-b were increased above the threshold value while the magnitude of phase-c MOV energy was found nearly zero. The same principle was used in fault classification method of this scheme as explained in Proposed Algorithm section.

The FSC protection scheme was found working effectively for all the fault simulations performed. Evidently, the design settings for FSC were found correct. At various locations, different types of faults were tested to find out the maximum deviation of the ANN estimated distance measured from the relay location, from the actual fault location. All the absolute errors were found to be well within 2 %. Tabulated results show that the soft computation technique of ANN captures the nonlinear relationship of input signals properly as evidenced by absolute error calculations. It can easily be seen from all the tables that the scheme is robust and accurate for varying fault conditions.

Fault classification accuracy aspect of the proposed algorithm has been compared with some of the methods in the literature in Table 8. Also, Fault location % absolute error aspect comparison of the proposed algorithm w.r.t fault location of other schemes is presented in Table 9.

**Table 8:** Fault classification comparision

References	FIA range (°)	Fault classification accuracy (%)
Parikh et al. (2008)	0–115	93.92
Vyas et al. (2014a, b)	0–85	99.39
Moravej et al. (2012)	0–360	99.21
Proposed method	0–360	100

**Table 9:** Fault Location Comparitive analysis

References	FIA range (°)	Compensated (yes/no)	Max. Abs. error (%)
Al-Mohammed and Abido (2014)	0–150	Yes	>2
Jain et al. (2009)	0–90	No	2.6
Reddy and Mohanta (2008)	0–180	No	6
Reddy and Mohanta (2007)	0–180	No	6.5
Meyar-Naimi (2012)	Not specified	Yes	10
Proposed method	0–360	Yes	<2

The overall superiority of the proposed algorithm is stated as follows:

- This algorithm requires only local per phase MOV energy measurement which is easily available at substation end.
- It does not need any feature extraction process from input signals which is mostly used in other algorithms in literature and hence the computational burden is reduced.
- No need of other end measurement, i.e. synchronized or unsynchronized data. So, the two end communication delay problems are avoided and cost is saved.
- MOV energy signals are not much affected as compared to impedance measurement signals used in other distance protection schemes due to fixed series compensation.
- The system considered in the proposed algorithm is modeled using real 400 kV grid parameters.

## Conclusions

A new online ANN–MOV energy based accurate scheme for fault detection, classification and fault location estimation in a series compensated transmission line has been proposed. The proposed algorithm provides a novel method for accurately estimating the fault location using only one end measurement where the FSC is installed. This novel scheme proves that it suits well in such fixed series compensated transmission system for complete protection application. The proposed algorithm has been suggested to the competent testing authority at the substation and the same has agreed to test run the algorithm with real time fault data during test commissioning.

The proposed scheme requires measurement data of MOV energy over only a short duration of post fault to classify and estimate the location of fault accurately. This fault location scheme gives estimates for fault distances which are well within the 2 % error margin with proposed high accuracy 0.2 class measuring devices mentioned. The proposed online scheme, with simplicity, high accuracy and fast performance, is a smart investment for power system protection application. Thus, this ANN based scheme is proposed as a potential solution to detect, classify and locate faults accurately in 400 kV series compensated line.

## Appendix

Transmission line parameters:

- Series compensation degree—40 %
- Line length—400 km
- Conductor type—quad aluminium conductor steel reinforced (ACSR) Moose
- Thermal limit of quad Moose conductor—3000 A
- *X*_*l*_ = 91.85 Ω (line reactance)
- *R*_*1*_ = 0.0146 Ω/km, *X*_1_ = 0.2530 Ω/km, *B*_1_ = 4.5777 micro-mho/km (positive sequence values)
- *R*_0_ = 0.2479 Ω/km, *X*_0_ = 1.0001 Ω/km, *B*_0_ = 2.6345 micro-mho/km (zero sequence values)

Real time data for Simulation:

- Bus Voltage level—400 kV
- Fault MVA level—2.45 GVA
- Frequency—50 Hz
- Total MOV columns in parallel—5 + 1 hot spare
- Reference current per MOV column—500 A
